# Prediction of 8-year risk of cardiovascular diseases in Korean adult population

**DOI:** 10.1038/s41598-021-93840-2

**Published:** 2021-07-12

**Authors:** Sung Hyouk Choi, Seung Min Lee, Su Hwan Kim, Minseon Park, Hyung-Jin Yoon

**Affiliations:** 1 Department of Biomedical Engineering, Seoul National University College of Medicine, 101 Daehakro, Jongno-gu, 03080 Seoul Korea; 2 Department of Family Medicine, Seoul National University Hospital, 101 Daehakro, Jongno-gu, 03080 Seoul Korea

**Keywords:** Cardiology, Medical research, Risk factors

## Abstract

Although many prediction models for cardiovascular diseases (CVDs) have been developed and validated for Western populations, the development of CVD prediction models for Asians has been slow. Our cohort study retrospectively analyzed the incidence of CVD that occurred between January 1, 2009, and December 31, 2016, in all Koreans who underwent national health screening. This dataset included 21,581,796 adults between the ages of 40 and 79 years (10,412,947 men, 11,168,849 women) without CVD at baseline. The primary outcome, CVD, was defined as the development of any of the following: acute coronary syndrome, cerebral infarction, and cerebral hemorrhage, as defined with health insurance claims data. The prediction model was constructed by Cox proportional hazard regression and validated with tenfold cross-validation. The performance of the models was evaluated through Harrell’s C-index and Brier score. The discrimination of the models was assessed by the area under the receiver operating characteristic curve (AUROC). Our model showed an AUROC of 0.762 in men and 0.811 in women. The Brier score of our model was 0.018 in men and 0.010 in women, which was better than the pooled cohort equation (PCE). Our novel model performed better than the FRS and PCE for Koreans.

## Introduction

Cardiovascular disease (CVD) is a major cause of death worldwide^1^, with 285.5 deaths per 100,000 people in 2015^2^. Because CVD is a heavy health burden, it is necessary to prevent CVD by identifying high-risk groups with risk prediction models that enable personal lifestyle modifications^3^.

Since the prediction model was developed using the Framingham Heart Study in 1976^4^, the prevalence and mortality of CVD have been declining in the United States^5^. Although the Framingham risk score (FRS) has been continuously updated until recently^6^, overestimation after applying the FRS to ethnic populations has been problematic in Western countries^7^.

To overcome the limitations of FRS, the pooled cohort equation (PCE) has been introduced by the American College of Cardiology/American Heart Association (ACC/AHA)^8^. The PCE predicts nonfatal myocardial infarction, fatal coronary heart disease, and nonfatal and fatal stroke in people aged 40–79 years and is publicly available with a simplified scoring sheet for primary care. The PCE has been validated not only in the United States^9^ but also in many other countries, including Asian countries^10,11^, and it was revised in 2018^12^.

Asians are at least two-thirds less likely to die from CVD^2^ than Americans. As Asian countries are already entering an aging society^13^ and CVD death and disablement are increasing^14^, a reliable CVD prediction model is necessary.

Previously developed models for Western populations have been criticized for overestimating the risk of CVD in Asian countries^15^. Accordingly, other research groups constructed prediction models and verified models in a study involving Koreans who underwent voluntary health screening^16^ and a study involving Chinese people enrolled in a prospective cohort^17^.

By applying the PCE based on whites and African-Americans to Asians, the predictive performance is expected to deteriorate because of over- or underestimation, depending on the risk group in Korea and China. The researchers suggested the following differences: the risks varied by demographics, duration of follow-up, and definition of outcomes according to the cohort. For these reasons, an improved CVD prediction model is needed that can be accurately applied to Asians.

The purpose of this study was to develop a model that predicts the 8-year risk of incident CVD for Asians using health screening data and health insurance claims data of the Korean population and to compare the performance of our model with the PCE.

## Results

Table 1 shows the baseline characteristics of the participants by sex. The mean age ± standard deviation was 51.5 ± 10.1 years for men and 52.3 ± 10.5 years for women. The person-years were 53,955,629 for men, 59,078,475 for women.

**Table 1:** Baseline characteristics of the subjects by sex.

Variables	Men (n = 10,412,947)	Women (n = 11,168,849)	*p*-value
Age (year)	51.5 ± 10.1	52.3 ± 10.5	< 0.0001
Systolic BP (mmHg)	125.6 ± 14.6	121.0 ± 15.7	< 0.0001
Diastolic BP (mmHg)	78.7 ± 10.1	75.0 ± 10.2	< 0.0001
Waist circumference (cm)	84.5 ± 7.8	77.4 ± 8.7	< 0.0001
Fasting serum glucose (mg/dL)	103.1 ± 29.1	97.1 ± 22.5	< 0.0001
Gamma-glutamyl transferase (U/L)	54.9 ± 71.6	24.1 ± 30.5	< 0.0001
Serum total cholesterol (mg/dL)	197.8 ± 37.0	200.1 ± 37.5	< 0.0001
HDL (mg/dL)	51.8 ± 13.1	58.0 ± 14.0	< 0.0001
LDL (mg/dL)	115.2 ± 35.3	118.8 ± 34.6	< 0.0001
Triglycerides (mg/dL)	156.9 ± 103.3	115.5 ± 71.1	< 0.0001
eGFR < 60 mL/min/1.73 m^2^	3.8%	4.8%	< 0.0001
Proteinuria^a^	3.0%	2.4%	< 0.0001
**Body mass index (kg/m**^**2**^**)**			< 0.0001
< 18.5	2.0%	3.3%	
≥ 18.5, < 25.0	58.2%	66.2%	
≥ 25.0, < 30.0	36.0%	26.3%	
≥ 30.0	3.8%	4.3%	
**Smoking**			< 0.0001
Nonsmoker	28.9%	94.1%	
Ex-smoker	28.1%	1.8%	
Smoker	42.9%	4.1%	
Pack-years (among ever-smokers)	11.7 ± 13.7	0.4 ± 2.7	< 0.0001
**Alcohol drinking**			< 0.0001
No drinking	34.4%	77.0%	
Low risk	57.3%	20.8%	
Moderate risk	5.2%	1.8%	
High risk	3.2%	0.5%	
**Activity group**			< 0.0001
Low activity	36.4%	44.4%	
Moderate activity	51.4%	46.3%	
High activity	12.2%	9.3%	
Patients with hyperlipidemia	2.0%	3.0%	< 0.0001
Patients with hypertension	16.3%	16.6%	< 0.0001
Patients with diabetes	6.8%	5.3%	< 0.0001
**Family history**			
Heart disease	3.3%	3.9%	< 0.0001
Stroke	6.5%	6.8%	< 0.0001
Incident CVD events	195,022 (1.9%)	111,546 (1.0%)	< 0.0001

Percentages for categorical variables; the mean ± standard deviation for continuous variables.

The *p*-value was calculated by t-test for continuous variables and Pearson’s chi-squared test for categorical variables.

*BP* blood pressure, *HDL* high-density lipoprotein cholesterol, *LDL* low-density lipoprotein cholesterol, *eGFR* estimated glomerular filtration rate, *CVD* cardiovascular disease.

^a^≥ 1 + in urine dipstick for proteinuria.

The proportion of the population who were smoking, drinking and performed moderate to high physical activity was higher in men than in women. The proportion of women with a family history of CVD was slightly higher than that of men.

The mean level of blood pressure, fasting serum glucose, serum triglycerides, and gamma-glutamyl transferase level was substantially higher in men, whereas the prevalence of hyperlipidemia and hypertension, serum total cholesterol, high-density lipoprotein cholesterol (HDL) and low-density lipoprotein cholesterol (LDL) levels were higher in women. Abnormal eGFR was more common in women than in men.

### CVD risk prediction model

During the 8-year follow-up period, a total of 195,022 CVD events occurred in men (1.9%) and 111,546 (1.0%) in women. The incidence density of CVD was 361.5 cases per 100,000 person-years among men and 188.8 cases per 100,000 person-years among women. Current smoking [men: adjusted hazard ratio (HR) 1.463, 95% confidence interval (CI) 1.443–1.484; women: HR 1.886, 95% CI 1.821–1.953], taking diabetes medicine (men: 1.414, 1.394–1.434; women: 1.504, 1.477–1.532), physical activity (men: 0.916, 0.907–0.924 in moderate-activity group; 0.910, 0.897–0.923 in high-activity group; women: 0.914, 0.902–0.925 in moderate-activity group; 0.893, 0.874–0.913 in high-activity group), and proteinuria (men: 1.409, 1.384–1.435; women: 1.446, 1.408–1.484) were significant (Table 2).

**Table 2:** Adjusted hazard ratios of the variables included in the CVD prediction model.

Variables	Men	Women
	Adjusted HR (95% CI)	Adjusted HR (95% CI)
Age (year)	1.061 (1.060–1.061)	1.074 (1.073–1.075)
Systolic BP (mmHg)	1.010 (1.010–1.010)	1.010 (1.010–1.011)
Diastolic BP (mmHg)	1.007 (1.006–1.008)	1.009 (1.008–1.010)
Waist circumference (cm)	1.004 (1.003–1.004)	1.006 (1.005–1.007)
Fasting serum glucose (mg/dL)	1.003 (1.003–1.003)	1.003 (1.003–1.004)
Gamma-glutamyl transferase (U/L)	1.001 (1.001–1.001)	1.001 (1.001–1.001)
Serum total cholesterol (mg/dL)	1.005 (1.005–1.005)	1.003 (1.002–1.004)
HDL (mg/dL)	0.988 (0.988–0.989)	0.991 (0.990–0.992)
LDL (mg/dL)	1.001 (1.000–1.001)	0.999 (0.998–1.000)
Triglycerides (mg/dL)	1.000 (1.000–1.000)	1.000 (1.000–1.001)
eGFR < 60 mL/min/1.73 m^2^	1.174 (1.156–1.192)	1.181 (1.161–1.202)
Proteinuria^a^	1.409 (1.384–1.435)	1.446 (1.408–1.484)
**Body mass index (kg/m**^**2**^**)**		
< 18.5	0.982 (0.951–1.014)	1.141 (1.095–1.188)
≥ 25.0, < 30.0	1.021 (1.009–1.033)	0.958 (0.944–0.973)
≥ 30.0	1.013 (0.984–1.042)	0.867 (0.841–0.895)
**Smoking**		
Ex-smoker	0.889 (0.876–0.901)	1.176 (1.113–1.242)
Smoker	1.463 (1.443–1.484)	1.886 (1.821–1.953)
Pack-year	1.005 (1.005–1.005)	1.004 (1.002–1.006)
**Alcohol drinking**		
Low risk	0.795 (0.787–0.803)	0.990 (0.971–1.010)
Moderate risk	0.760 (0.743–0.777)	1.126 (1.064–1.191)
High risk	0.783 (0.762–0.804)	1.154 (1.049–1.269)
**Activity group**		
Moderate activity	0.916 (0.907–0.924)	0.914 (0.902–0.925)
High activity	0.910 (0.897–0.923)	0.893 (0.874–0.913)
**Current medication**		
Blood glucose-lowering drugs	1.414 (1.394–1.434)	1.504 (1.477–1.532)
Antihypertensive drugs	1.183 (1.170–1.196)	1.240 (1.223–1.257)
Lipid-modifying drugs	0.996 (0.969–1.023)	0.822 (0.798–0.846)
Family history of heart disease	1.331 (1.297–1.365)	1.076 (1.037–1.117)
Family history of stroke	1.167 (1.146–1.187)	1.159 (1.133–1.186)

S_0_(8) of men = 0.9798, S_0_(8) of women = 0.9916. Covariates included in Cox models were selected by stepwise procedures.

*HR* hazard ratio, *CI* confidence interval, *BP* blood pressure, *HDL* high-density lipoprotein cholesterol, *LDL* low-density lipoprotein cholesterol, *eGFR* estimated glomerular filtration rate.

^a^≥ 1 + in urine dipstick for proteinuria.

In the analysis of smoking, the past smoker category was protective against CVD in men (0.889, 0.876–0.901) but not in women (1.176, 1.113–1.242). Among women, the risk of CVD increased as the amount of alcohol ingestion increased (moderate risk: 1.126, 1.064–1.191, high risk: 1.154, 1.049–1.269). However, for men, the risk of CVD did not increase with any level of alcohol consumption. The family history of heart disease and stroke was associated with an increased risk of CVD in both sexes (Table 2).

For men, the risk of CVD increased when the BMI was in the overweight category (1.021, 1.009–1.033). However, women were at the most hazardous risk in the underweight category (1.141, 1.095–1.188), and the risk of CVD decreased in the overweight (0.958, 0.944–0.973) and obesity categories (0.867, 0.841–0.895; Table 2).

When comparing our proposed CVD prediction model to the extended Cox model, the coefficient of proteinuria increased in both men (from 1.409, 1.384–1.435 to 1.512, 1.485–1.539) and women (from 1.446, 1.408–1.484 to 1.573, 1.533–1.614). However, there were no noticeable changes in the other variables (Supplementary Tables 1, 2 and 3).

### Performance of the CVD prediction model

**Figure 1:**
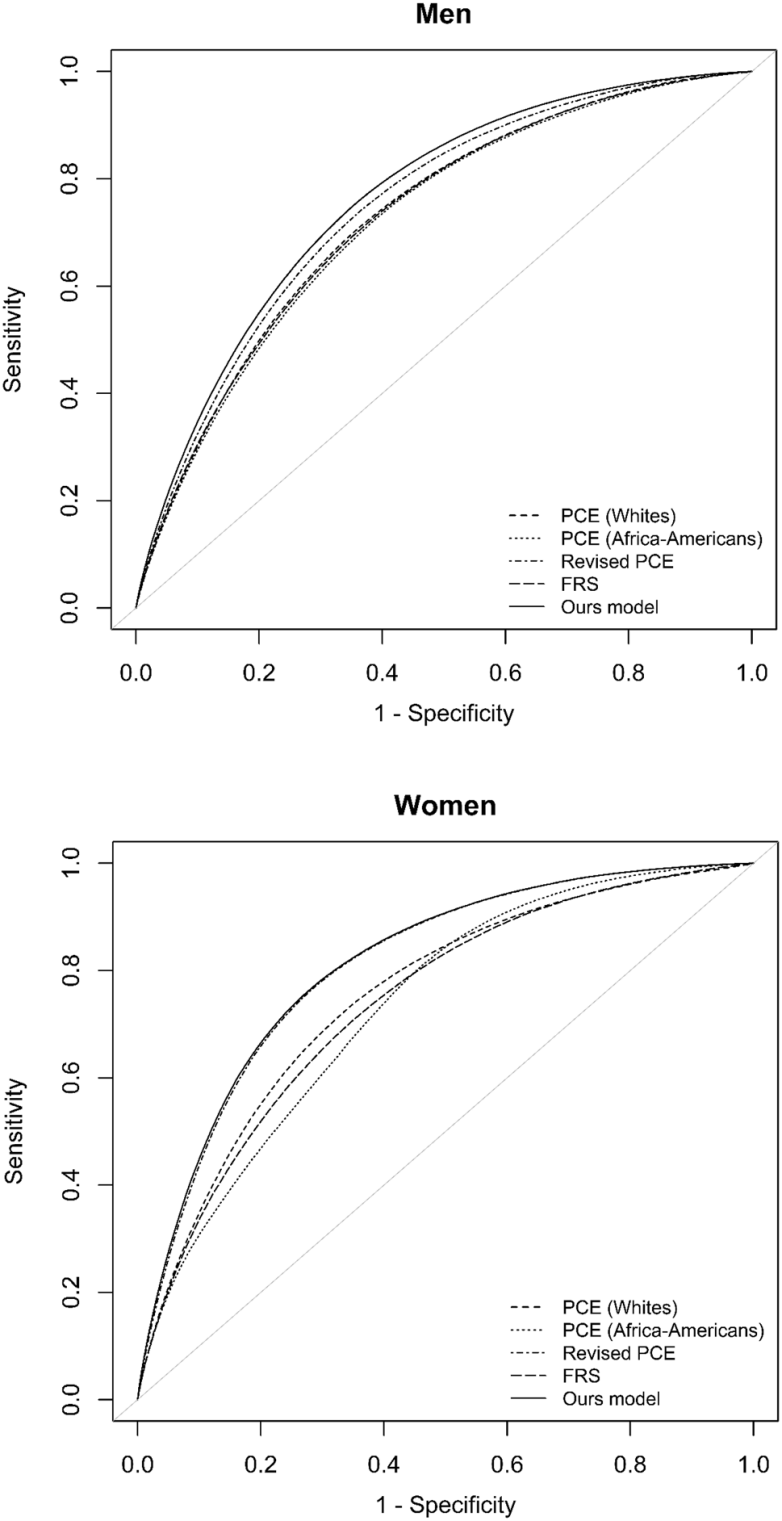
Comparison of the area under the receiver operating characteristic curve (AUROC) of the Framingham risk score (FRS), pooled cohort equation (PCE), and our model.

Table 3 summarizes the performance of the CVD risk prediction model for both sexes. The sex-specific prediction model performed well on both model discrimination parameters. The average Harrell’s C in the validation for our model was 0.749 ± 0.002 in men and 0.795 ± 0.002 in women. The cross-validation results of the AUROC are shown in Fig. 1. Compared to the AUROC of the revised PCE, the AUROC of our model improved from 0.748 ± 0.002 to 0.762 ± 0.002 in men and from 0.808 ± 0.002 to 0.811 ± 0.002 in women. For comparison, other models had an AUROC ranging from 0.723 ± 0.002 to 0.730 ± 0.002 for men and 0.734 ± 0.001 to 0.753 ± 0.002 for women. In terms of the Brier score, our model showed the best performance, with 0.018 in men and 0.010 in women. In comparison, the other models had values of 0.024 to 0.032 in men and 0.011 to 0.025 in women.

**Table 3:** Results of tenfold cross-validation of Harrell’s C-index, the area under the receiver-operator characteristic curve (AUROC), Brier score for the pooled cohort equation (PCE), and our model.

	AUROC	Harrell’s C	Brier score
**Men**			
Framingham risk score	0.728 ± 0.002	0.715 ± 0.003	0.032 ± 0.003
PCE (whites)	0.730 ± 0.002	0.715 ± 0.003	0.031 ± 0.000
PCE (African-Americans)	0.723 ± 0.002	0.710 ± 0.003	0.026 ± 0.000
Revised PCE	0.748 ± 0.002	0.737 ± 0.002	0.024 ± 0.000
Our model	0.762 ± 0.002	0.749 ± 0.002	0.018 ± 0.000
**Women**			
Framingham risk score	0.741 ± 0.001	0.723 ± 0.002	0.013 ± 0.000
PCE (whites)	0.753 ± 0.002	0.734 ± 0.003	0.016 ± 0.000
PCE (African-Americans)	0.734 ± 0.001	0.712 ± 0.001	0.025 ± 0.000
Revised PCE	0.808 ± 0.002	0.792 ± 0.002	0.011 ± 0.000
Our model	0.811 ± 0.002	0.795 ± 0.002	0.010 ± 0.000

The mean ± standard deviation.

## Discussion

We developed a model to predict CVD using the nationwide health screening data of Korea, which showed better performance than PCE in Koreans. This model included predictor variables such as lifestyle, family history, and blood/urine test conducted in the screening for all Koreans. Since the results of the studies that we compared were based on voluntary participants, it was possible that there may be a difference in the composition of the total population and the results of screenings and questionnaires related to CVD. However, our study predicted outcomes with data from a large number of Koreans, and it can be said that the data represent the overall pattern of Koreans.

The association of atherosclerosis in the coronary arteries with that in the cerebral vasculature is well known^18^. Blood pressure, smoking, serum concentrations of total cholesterol, HDL, and LDL have been discussed as modifiable risk factors for atherosclerosis^19^. Furthermore, clinical data support that these factors are clustered and multiplicatively interactive. Therefore, developing predictive models for composite outcomes and merging coronary artery and cerebrovascular disease have been advocated to estimate global risk and to modify risk factors.

The risk predicted by the interaction of these risk factors may vary depending on the characteristics of the population subgroups. In this study, the association of smoking, alcohol consumption, and BMI with the risk of CVD differed by sex. Among current smokers, women were at a higher CVD risk than men, which is supported by a meta-analysis of 75 cohort studies demonstrating a 25% greater risk for coronary heart disease in female smokers than in male smokers^20^. In the case of past smokers, previous studies have shown that although smoking cessation at any age significantly reduces the morbidity of CVD, women were still at increased risk of CVD after quitting smoking^21^. Perhaps a longer smoking cessation period will result in greater benefits, but the follow-up period of our study may not be long enough to observe the beneficial effects of smoking cessation.

Both sexes showed different patterns of BMI effects on CVD. Men were protected against CVD, although this relationship was not significant in the underweight category compared to normal BMI. However, women were most at risk in the underweight category compared to normal BMI. In addition, women were protected against CVD as BMI increased. There are several possible explanations for this observation^22^, and women are more likely to be metabolically healthy obese (MHO). The definition of MHO varies across studies; using strict criteria of neither metabolic syndrome components nor any previous CVD diagnosis, a recent cohort study reported that the prevalence of MHO varied from 3.3 to 32.1% in men and from 11.4 to 43.3% in women among obese participants^23^. Further studies are necessary to prove whether the sex difference is actually due to MHO. In the alcohol consumption analysis, women were at increased risk of CVD when they consumed moderate- to high-risk doses; however, in men, alcohol consumption was protective across all categories. The variations in the risk group of alcohol consumption between sexes are likely to result from differences in biological and psychosociocultural susceptibility to alcohol between sexes^24^.

Many studies in different countries predict the risk of CVD with different methodologies and diverse variables^25,26^. Recently, the PCE ethnic- and sex-specific prediction model, based on 5 major epidemiological studies of the United States, was developed to predict the risk of CVD development within 10 years, depending on age, total cholesterol, HDL, systolic blood pressure, smoking, and diabetes mellitus. The model has been continuously validated^15^, and validation has been conducted in Asian countries among Koreans and Chinese^17^. However, the PCE overestimated the CVD risk in the Korean population with the Korean Heart Study (KHS)^16^. According to the survey of a total of 192,605 persons (114,622 men and 77,983 women), the absolute 10-year CVD risk for men in the KHS cohort was overestimated by 56.5% (whites’ model) and 74.1% (African-American model), while the risk for women was underestimated by 27.9% (whites) and overestimated by 29.1% (African-Americans). Additionally, a Korean risk prediction model for atherosclerotic cardiovascular disease was developed in that study. During the mean 12.8 years of the follow-up period, there were 12,327 cases of CVD. The AUROC of that prediction model was 0.741 for men and 0.745 for women. When the authors applied the same cohort data to PCE, they obtained slightly lower AUROCs: 0.727 (whites) and 0.725 (African-Americans) in men and 0.738 (whites) and 0.739 (African-Americans) in women. Although the AUROC is similar in both models, the incidence of CVD is overestimated and is more than twice as high as the observed CVD events, especially in Korean men. This observation emphasizes the necessity of a risk estimation model for the Asian population rather than applying models developed for Western populations.

In our study, the incidence of CVD was 1.9% in men and 1.0% in women over 8 years of follow-up, and depending on the cohort used for data analysis of the PCE, the incidence of 10-year CVD by ethnicity and sex varied between 1.0 and 28.5% in men and 0.0% and 23.0% in women ^8^. The United States has many ethnicities in the country, and the risk of CVD is lower in Hispanic and Asian Americans than in non-Hispanic Americans and higher in American Indians. Asians also comprise multiple ethnicities, and health characteristics such as lifestyle are different^2^, so it can be expected that the risk of CVD is different between ethnic groups. Because the absolute risk of CVD observed in Asian countries such as Korea is quite different from that observed in Western countries, Asians need a CVD risk prediction model of their own.

In the development of our Cox model, we explored the proportional hazard assumption which is one of the essential aspects of the Cox proportional hazard model. Schoenfeld residuals were plotted over time and the slope of the fitted line was tested. Based on the *p*-values provided from the test, most variables used in our model had very small *p*-values indicating a violation of the proportional hazard assumption. However, this is a Chi-squared test that is known to be very sensitive in large samples^27^. For this reason, Klein and Moeschberger suggested a graphical approach^28^. Supplementary Figs. 1 and 2 (Schoenfeld residuals) show that fitted lines are close to horizontal near y = 0 indicating that the covariates follow proportional hazard assumption. Additionally, the time-varying (extended) Cox model was fitted to further verify the proportional hazard assumption and the results are presented in Table 3 in the supplementary document. The results show that there was no drastic change in coefficients and directions of the estimated hazard ratios from the proposed CVD prediction model indicating that the covariate-time interactions are not present. Based on these results, we decided to use our Cox proportional hazard model over the extended Cox model for the following reasons: (1) The proportional hazard assumptions are met. (2) The extended model does not improve predictive power or statistical efficiency. (3) It is not practical in clinical practice.

Although we used a large dataset, this study has some limitations. First, although the CVD outcomes of our study were supplemented by further criteria in addition to ICD-10 codes, the possibility of overreporting could not be excluded. Second, although health screening is mandatory in Korea, the underrepresentation of some vulnerable groups might be an issue in our study. The population of our study, however, was free of CVD at baseline, and the bias caused by the underrepresentation of vulnerable groups must be minimal. Notwithstanding the limitations mentioned above, there are merits to our study. First, our prediction model was based on the comprehensive health screening data of a cohort with a large sample size (n = 21,581,796), covering a substantial proportion of the entire Korean population. All the data were collected using a standardized protocol before the development of the results of interest. Second, in addition to the variables of the PCE, our model incorporated the effects of lifestyle such as physical activity, alcohol consumption, smoking status, and personal and family history of CVD-related diseases.

We developed a novel model to predict CVD by using nationwide health screening data from Korea, which performed better than the PCE for Koreans. Future studies are necessary to validate our model in other Asian countries and to test its clinical utility.

## Methods

This study was approved by the Institutional Review Board (IRB) of Seoul National University, Seoul, Korea (1606-016-768). The requirement for informed consent and approval was waived by IRB because of the nature of this study, which retrospectively analyzed the national registry data. All methods and procedures were carried out in accordance with the relevant guidelines and regulations.

### Study population

All Korean citizens are covered by a single insurance system, and all claims data are digitized and collected by the National Health Insurance Service (NHIS). The NHIS also collects the national health screening data, which is mandatory every year or every 2 years, free of charge, from all screening hospitals^29^, and in 2014, the participation rate of the program among the eligible population was 74.8%^30^.

**Figure 2:**
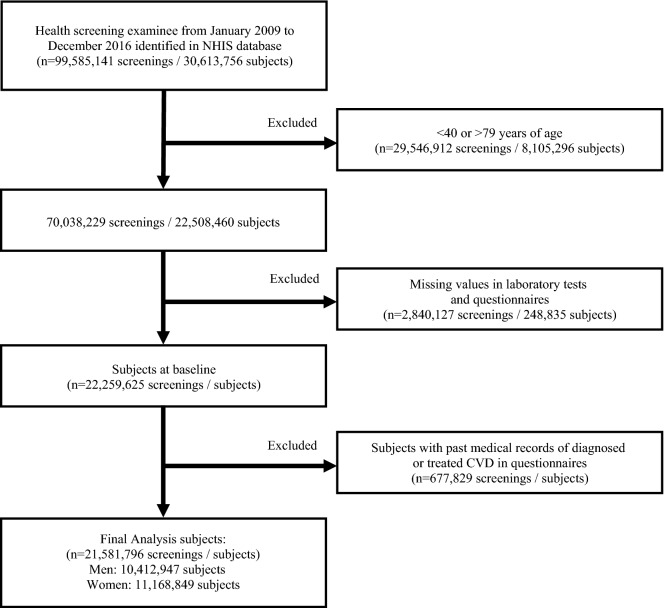
Defining the study population.

Our study covered 99,585,141 health screenings performed on 30,613,756 subjects between January 2009 and December 2016. We excluded the data of 29,546,912 health screenings performed on subjects who were < 40 years or > 79 years at the time of health screening. The data of 2,840,127 health screenings with missing values in questionnaires and laboratory tests were excluded. Among 22,259,625 subjects, 677,829 subjects with past medical records of diagnosed or treated CVD in the self-reported standard questionnaires at baseline were excluded. Finally, the baseline health screening data of 21,581,796 subjects aged 40–79 were analyzed (Fig. 2). According to the 2017 Population and Housing Census by Statistics Korea^31^, there were approximately 25.7 million persons aged 40–79 years in Korea (12.6 million men and 13.0 million women). The study population of our study was equivalent to 84.0% of this age group.

The health screening data contained (1) demographic variables such as age, sex, and residence area; (2) variables regarding health behavior such as cigarette smoking status/dose/duration, frequency per week and amount per day of alcohol consumption, as well as the number of days per week of physical activity, medical history, and family history; (3) clinical measurement results such as height, body weight, blood pressure, and waist circumference; and (4) laboratory test results such as fasting serum glucose, lipid profile, hemoglobin, serum creatinine, liver enzyme, and urine dipstick test for proteinuria.

### Definition of the variables

Subjects were categorized as never, past, and current smokers according to their smoking status. The amount of cigarette smoking in the past and current smokers was measured by the number of pack years. We calculated the daily average alcohol consumption by the following equation according to the definition of alcohol consumption by World Health Organization (WHO)^32^: Daily average alcohol consumption (g/day) = [Alcohol consumption frequency (drinking days in a week) * Quantity (drinks per day) * Volume of glass * Alcohol by volume (0.2) * Density of alcohol (0.785)]/7 (days/week), where the standard drink was a glass of “Soju (distilled liquor commonly consumed in Korea)” (50 ccs). Then, the average alcohol consumption was classified into four categories as follows: (1) abstinent for alcohol; (2) low-risk drinking: average alcohol consumption < 40.0 g/day for men and < 20.0 g/day for women; (3) moderate-risk drinking: average alcohol consumption between 40.0 and 59.9 g/day for men and 20.0–39.9 g/day for women; and (4) high-risk drinking: average alcohol consumption $\ge$ 60.0 g/day for men and $\ge$ 40.0 g/day for women.

Regarding physical activity, the questionnaires asked for the frequency of vigorous-intensity movement at least 20 min per day (e.g., running, aerobics, fast biking, or climbing), of moderate-intensity movement at least 30 min per day (e.g., fast walking, doubles tennis, bike riding at common speed, or mopping), and of walking at least 30 min per day in the past seven days. Following the recommended scoring protocol of the International Physical Activity Questionnaire^33^, total physical activity metabolic equivalent task minutes per week (METs * min/week) was calculated by summing the frequency of walking (3.3 MET), moderate-intensity activity (4.0 MET), and vigorous-intensity activity (8.0 MET). We categorized physical activity into three levels as Victoria et al.^34^ proposed: low (< 600 METs * min/week), moderate (600–2999 METs * min/week), and high ($\ge$ 3000 METs * min/week) activity groups.

Body mass index (BMI) was categorized into four groups using the BMI classification by WHO^35^: underweight, < 18.5 kg/m^2^; healthy weight, 18.5–24.9 kg/m^2^; overweight, 25.0–29.9 kg/m^2^; and obesity, $\ge$ 30.0 kg/m^2^.

The estimated glomerular filtration rate (eGFR) was calculated using the Chronic Kidney Disease Epidemiology Collaboration equation^36^ based on the serum creatinine level, and abnormal eGFR was defined when eGFR was < 60 mL/min/1.73 m^2^. The results of the urine dipstick test for proteinuria were reported as negative, trace, and 1 + to 4 +, and proteinuria was defined as 1 + or higher.

### Definition of outcome

The outcome events were defined as CVD, including acute coronary syndrome (ACS), cerebral infarction, and cerebral hemorrhage. ACS was defined as I20 or I21 by the tenth revision of the International Classification of Disease^37^ (ICD-10) codes supplemented with a record of percutaneous coronary intervention, coronary artery bypass graft, or the prescription of thrombolytics in the claims data. Cerebral infarction was defined as I63 with a record of brain magnetic resonance image and the prescription of acetylsalicylic acid in the claims data. Cerebral hemorrhage was defined as I60, I61, or I62 with a record of brain computed tomography.

### Statistical analysis

The CVD prediction model was developed by using the Cox proportional hazards regression model. The proportional hazard assumption was tested through statistical tests and graphical diagnosis based on the Schoenfeld residuals (Supplementary Figs. 1 and 2). To compare the time-varying effect in our Cox model, the extended Cox model was developed and tested using the participants’ additional health screening data up to 5 times. (Supplementary Table 1). Covariates included in the models were selected by stepwise procedures. The tenfold cross-validation technique was used for model construction and validation. Harrell’s C-index was calculated to assess the performance of the prediction model. To assess the discrimination between our model and PCE, we calculated an area under the receiver-operating characteristic curve (AUROC) and Brier score. To match the baseline survival function closely, we divided the risk probability of PCE by 1.25 to obtain the 8-year estimated risk in our data^38^. We used R software (version 3.5.2; R foundation for Statistical Computing, Vienna, Austria) to conduct all statistical analyses. Two-sided *p* < 0.05 was considered significant.

## Supplementary Information

Supplementary Information 1.
