# Supplementary material for: Prediction of 8-year risk of cardiovascular diseases in Korean adult population
Source: Sci Rep. 2021 Jul 12;11:14339. doi: 10.1038/s41598-021-93840-2 (PMC8275773; doi:10.1038/s41598-021-93840-2)
Supplement: Supplementary file 1 — Supplementary Information 1. [file 41598_2021_93840_MOESM1_ESM.pdf]

SUPPLEMENTARY FIGURES

Supplementary Figure 1. Plots of Schoenfeld residuals of coefficients versus time for the sex-specific Cox model (men)

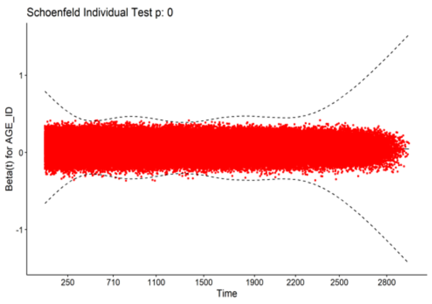

Age (p value <.0001)

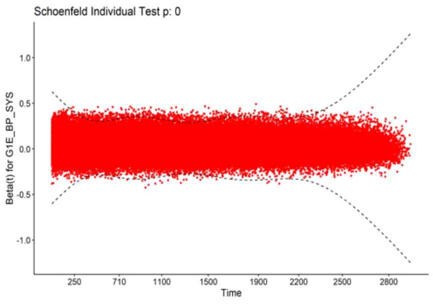

Systolic BP (p value <.0001)

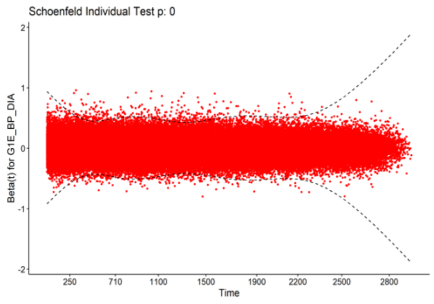

Diastolic BP (p value <.0001)

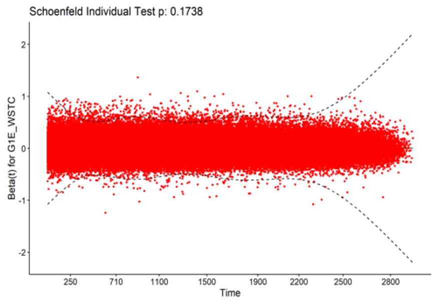

Waist circumference

(p value = 0.174)

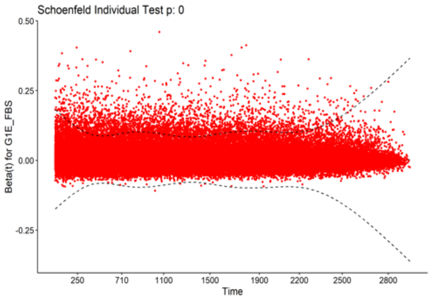

Fasting serum glucose

(p value <.0001)

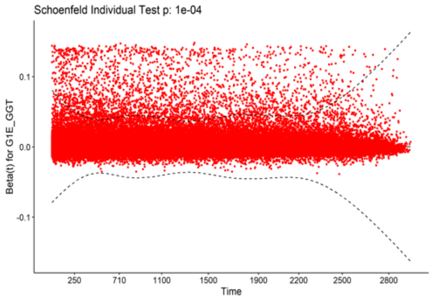

Gamma-glutamyl transferase

(p value <.0001)

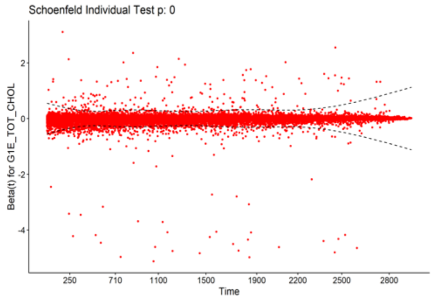

Serum total cholesterol

(p value <.0001)

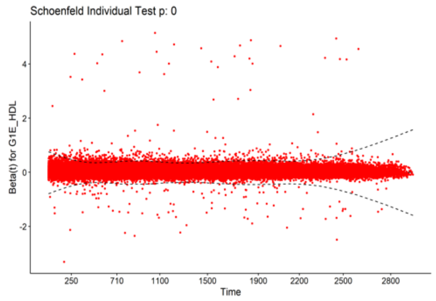

HDL (p value <.0001)

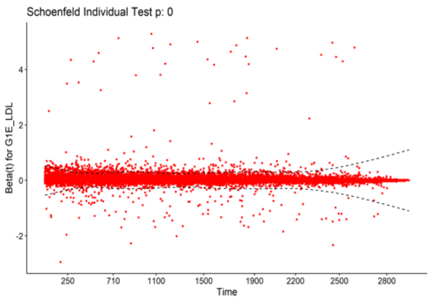

LDL (p value <.0001)

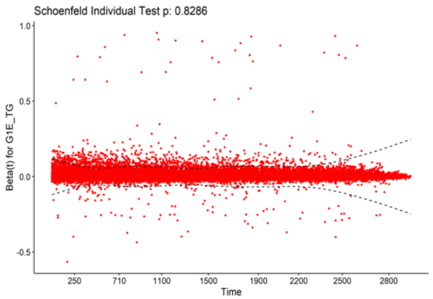

Triglycerides (p value = 0.829)

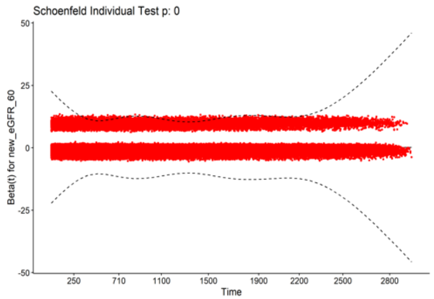

eGFR<60 mL/min/1.73m^2

(p value <.0001)

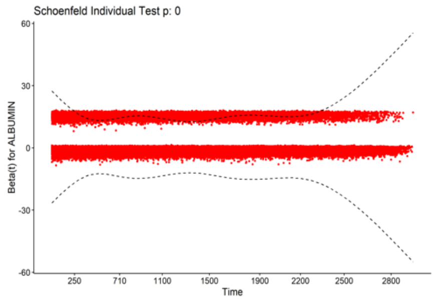

Proteinuria (p value <.0001)

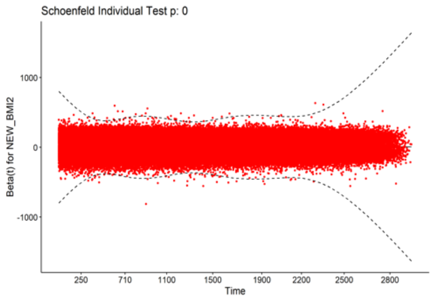

BMI (p value <.0001)

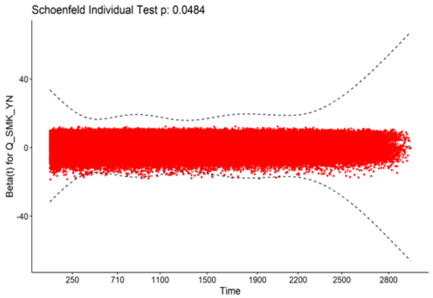

Smoking (p value = 0.048)

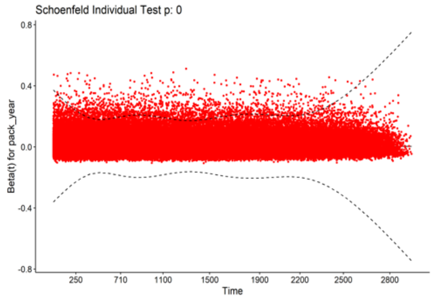

Pack-year (p value <.0001)

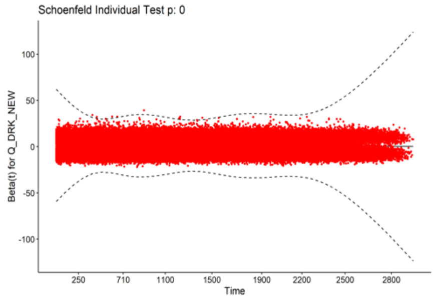

Alcohol (p value <.0001)

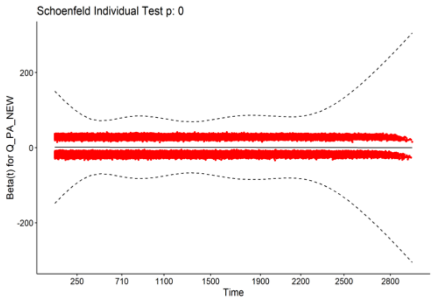

Activity (p value <.0001)

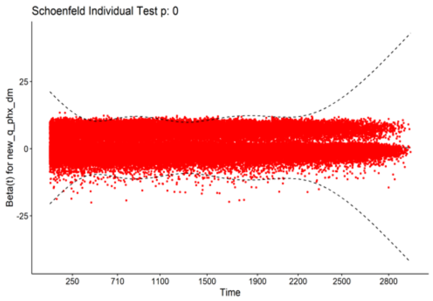

Current medication (Blood glucose lowering drugs) (p value <.0001)

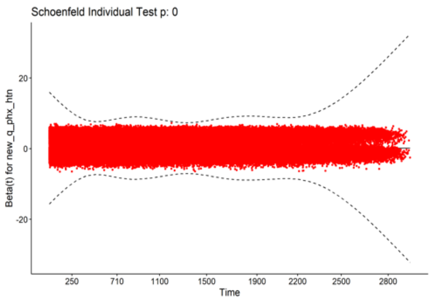

Current medication (Antihypertensive drugs) (p value <.0001)

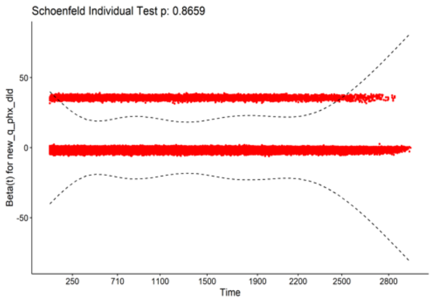

Current medication (Lipid-modifying drugs) (p value = 0.866)

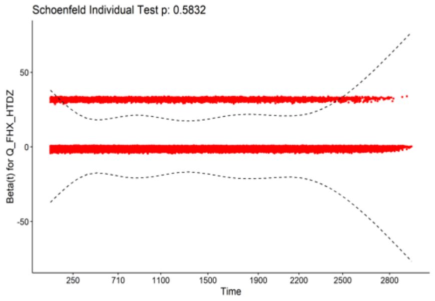

Family history of heart disease (p value = 0.583)

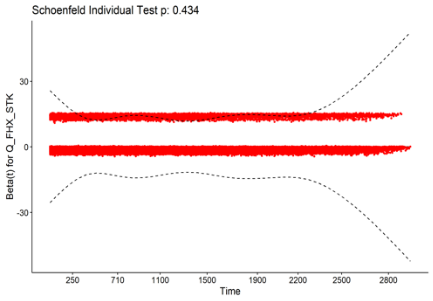

Family history of stroke (p value = 0.434)

Supplementary Figure 2. Plots of Schoenfeld residuals of coefficients versus time for the sex-specific Cox model (women)

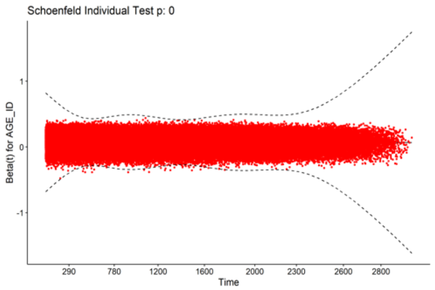

Age (p value <.0001)

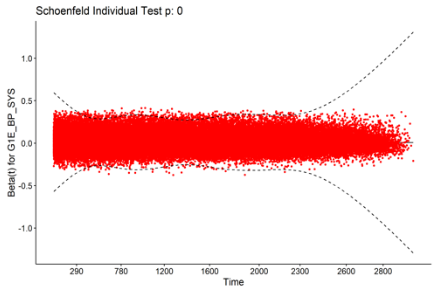

Systolic BP (p value <.0001)

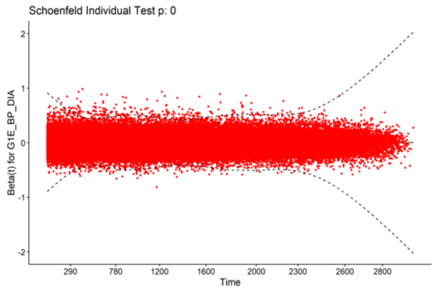

Diastolic BP (p value <.0001)

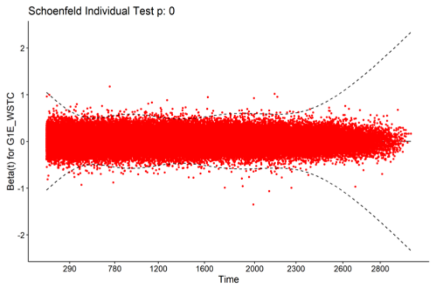

Waist circumference

(p value <.0001)

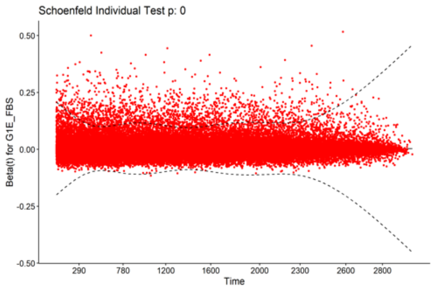

Fasting serum glucose

(p value <.0001)

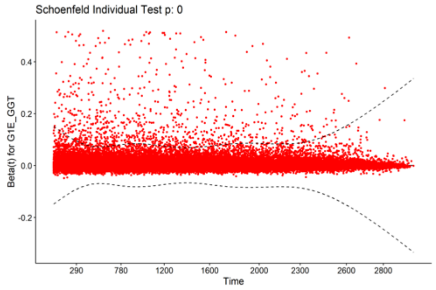

Gamma-glutamyl transferase

(p value <.0001)

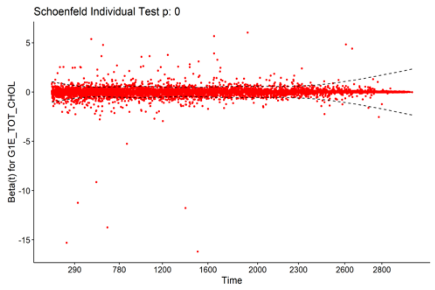

Serum total cholesterol

(p value <.0001)

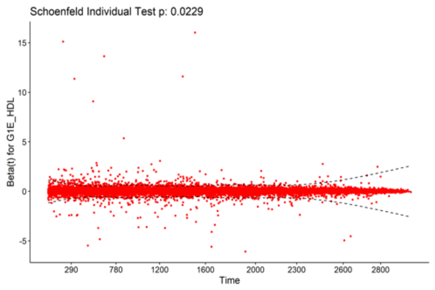

HDL (p value = 0.023)

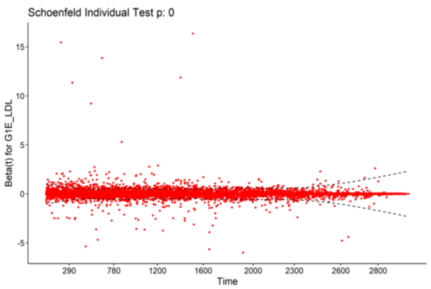

LDL (p value <.0001)

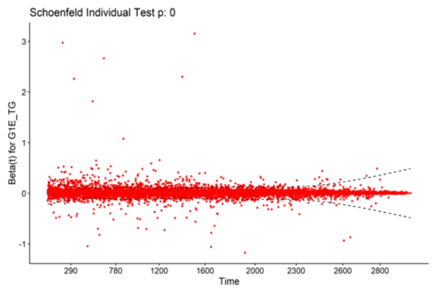

Triglycerides (p value <.0001)

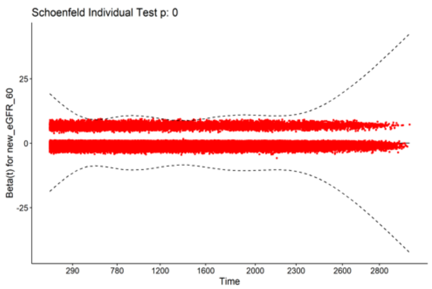

eGFR<60 mL/min/1.73m^2

(p value <.0001)

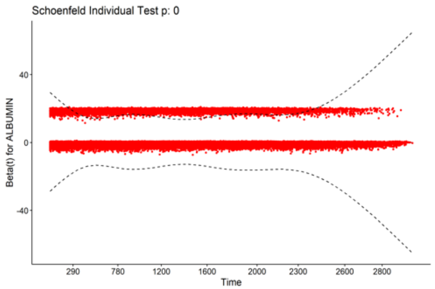

Proteinuria (p value <.0001)

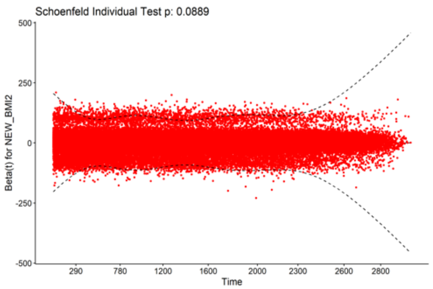

BMI (p value = 0.089)

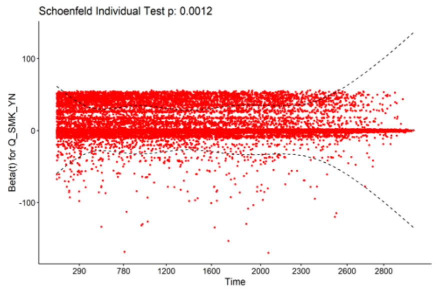

Smoking (p value = 0.001)

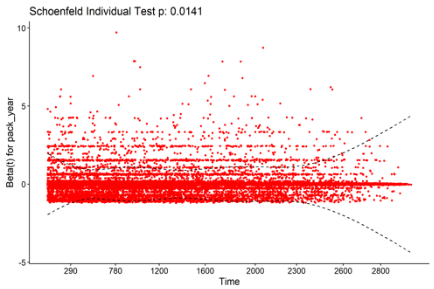

Pack-year (p value = 0.014)

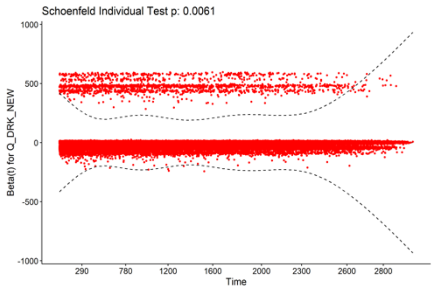

Alcohol (p value = 0.006)

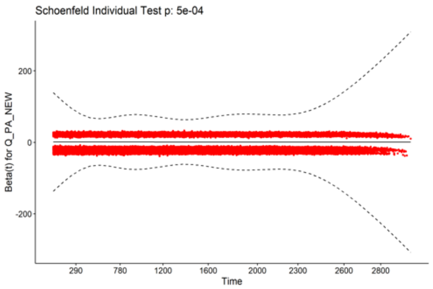

Activity (p value <.0001)

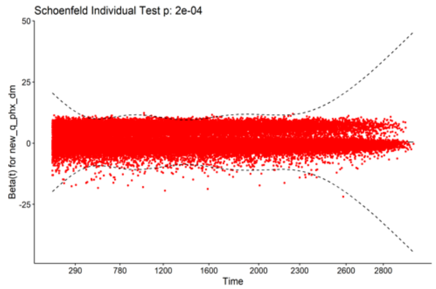

Current medication (Blood glucose lowering drugs) (p value <.0001)

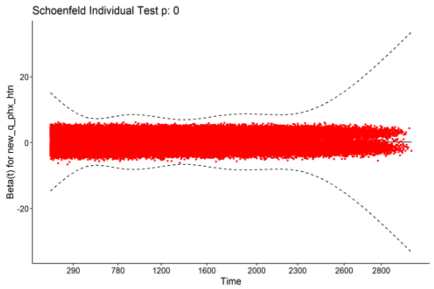

Current medication (Antihypertensive drugs) (p value <.0001)

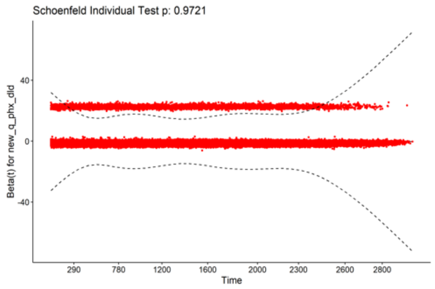

Current medication (Lipid-modifying drugs) (p value = 0.972)

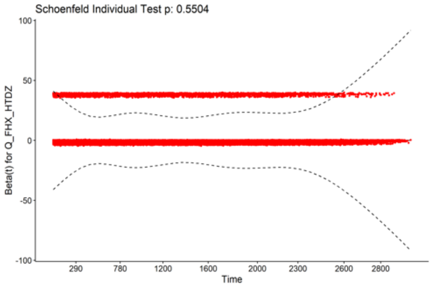

Family history of heart disease (p value = 0.550)

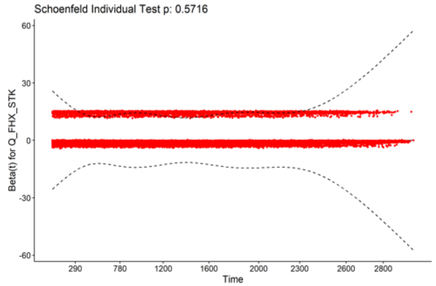

Family history of stroke (p value = 0.572)

SUPPLEMENTARY TABLES

Supplementary Table 1. Adjusted hazard ratios of the variables included in the extended Cox model

Variables	Men	Women
	Adjusted HR (95% CI)	Adjusted HR (95% CI)
Age (year)	1.062 (1.061-1.063)	1.075 (1.075-1.076)
Systolic BP (mmHg)	1.011 (1.011-1.011)	1.012 (1.011-1.012)
Diastolic BP (mmHg)	1.007 (1.006-1.008)	1.010 (1.009-1.011)
Waist circumference (cm)	1.003 (1.002-1.004)	1.005 (1.004-1.006)
Fasting serum glucose (mg/dL)	1.003 (1.003-1.003)	1.003 (1.003-1.004)
Gamma-glutamyl transferase (U/L)	1.001 (1.000-1.001)	1.001 (1.001-1.002)
Serum total cholesterol (mg/dL)	1.005 (1.005-1.006)	1.004 (1.003-1.005)
HDL (mg/dL)	0.985 (0.985-0.986)	0.989 (0.988-0.990)
LDL (mg/dL)	1.000 (1.000-1.001)	0.999 (0.998-1.000)
Triglycerides (mg/dL)	1.000 (1.000-1.000)	1.000 (1.000-1.000)
eGFR<60 mL/min/1.73 m^2	1.187 (1.169-1.206)	1.194 (1.174-1.215)
Proteinuria[a]	1.512 (1.485-1.539)	1.573 (1.533-1.614)
Body mass index (kg/m^2)		
<18.5	0.995 (0.963-1.027)	1.186 (1.141-1.234)
≥25.0, <30.0	1.006 (0.994-1.017)	0.948 (0.933-0.962)
≥30.0	1.005 (0.977-1.034)	0.852 (0.826-0.879)
Smoking		
Ex-smoker	0.890 (0.878-0.903)	1.199 (1.135-1.267)
Smoker	1.456 (1.435-1.476)	1.913 (1.847-1.982)
Pack-year	1.005 (1.005-1.005)	1.004 (1.002-1.006)
Alcohol drinking		
Low risk	0.803 (0.795-0.811)	0.992 (0.972-1.011)
Moderate risk	0.780 (0.763-0.797)	1.138 (1.076-1.204)
High risk	0.808 (0.787-0.830)	1.178 (1.072-1.296)
Activity group		
Moderate activity	0.909 (0.900-0.917)	0.904 (0.893-0.915)
High activity	0.898 (0.885-0.911)	0.877 (0.858-0.897)
Current medication		
Blood glucose-lowering drugs	1.394 (1.375-1.414)	1.515 (1.487-1.542)
Antihypertensive drugs	1.181 (1.169-1.194)	1.245 (1.229-1.262)
Lipid-modifying drugs	0.972 (0.946-0.999)	0.801 (0.778-0.824)

Family history of heart disease	1.336 (1.303-1.371)	1.076 (1.037-1.117)
Family history of stroke	1.167 (1.147-1.188)	1.155 (1.129-1.183)

$S_0(8)$ of men = 0.9798, $S_0(8)$ of women = 0.9916. Covariates included in Cox models were selected by stepwise procedures.

HR, hazard ratio; CI, confidence interval; BP, blood pressure; HDL, high-density lipoprotein cholesterol; LDL, low-density lipoprotein cholesterol; eGFR, estimated glomerular filtration rate

[a]≥1+ in urine dipstick for proteinuria.

Supplementary Table 2. Chi-square value and p-value of CVD prediction model

Variables	Men			Women		
	df	Chi-square	P-value	df	Chi-square	P-value
Age (year)	1	52241.770	<.0001	1	41162.186	<.0001
Systolic BP (mmHg)	1	2129.351	<.0001	1	1473.649	<.0001
Diastolic BP (mmHg)	1	471.399	<.0001	1	479.748	<.0001
Waist circumference (cm)	1	75.678	<.0001	1	133.694	<.0001
Fasting serum glucose (mg/dL)	1	2666.102	<.0001	1	1335.252	<.0001
Gamma-glutamyl transferase (U/L)	1	338.417	<.0001	1	350.305	<.0001
Serum total cholesterol (mg/dL)	1	664.332	<.0001	1	49.742	<.0001
HDL (mg/dL)	1	2017.570	<.0001	1	287.448	<.0001
LDL (mg/dL)	1	7.207	<.0001	1	3.446	0.063
Triglycerides (mg/dL)	1	0.562	0.453	1	11.142	0.001
eGFR<60 mL/min/1.73 m^2	1	423.001	<.0001	1	363.726	<.0001
Proteinuria[a]	1	1337.072	<.0001	1	756.070	<.0001
Body mass index (kg/m^2)						
<18.5	1	1.229	0.268	1	40.239	<.0001
≥25.0, <30.0	1	11.754	0.001	1	30.453	<.0001
≥30.0	1	0.721	0.396	1	78.838	<.0001
Smoking						
Ex-smoker	1	264.124	<.0001	1	33.362	<.0001
Smoker	1	2838.992	<.0001	1	1255.534	<.0001
Pack-year	1	1005.263	<.0001	1	18.300	<.0001
Alcohol drinking						
Low risk	1	2034.126	<.0001	1	1.037	0.308
Moderate risk	1	586.868	<.0001	1	17.065	<.0001
High risk	1	318.656	<.0001	1	8.704	0.003
Activity group						
Moderate activity	1	321.094	<.0001	1	203.536	<.0001
High activity	1	169.663	<.0001	1	103.508	<.0001
Current medication						
Blood glucose-lowering drugs	1	2277.645	<.0001	1	1940.170	<.0001
Antihypertensive drugs	1	932.099	<.0001	1	974.284	<.0001
Lipid-modifying drugs	1	0.099	0.753	1	175.497	<.0001
Family history of heart	1	477.716	<.0001	1	15.151	<.0001

disease						
Family history of stroke	1	299.543	<.0001	1	156.294	<.0001

BP, blood pressure; HDL, high-density lipoprotein cholesterol; LDL, low-density lipoprotein cholesterol; eGFR, estimated glomerular filtration rate

[a]≥1+ in urine dipstick for proteinuria.

Supplementary Table 3. Chi-square value and p-value of extended Cox model

Variables	Men			Women		
	df	Chi-square	P-value	df	Chi-square	P-value
Age (year)	1	52555.092	<.0001	1	40267.906	<.0001
Systolic BP (mmHg)	1	2623.143	<.0001	1	1999.573	<.0001
Diastolic BP (mmHg)	1	465.172	<.0001	1	552.518	<.0001
Waist circumference (cm)	1	64.584	<.0001	1	119.794	<.0001
Fasting serum glucose (mg/dL)	1	3008.243	<.0001	1	1361.690	<.0001
Gamma-glutamyl transferase (U/L)	1	363.384	<.0001	1	445.855	<.0001
Serum total cholesterol (mg/dL)	1	490.968	<.0001	1	62.826	<.0001
HDL (mg/dL)	1	2547.729	<.0001	1	371.748	<.0001
LDL (mg/dL)	1	1.585	0.2081	1	4.171	0.0411
Triglycerides (mg/dL)	1	15.555	<.0001	1	6.337	0.0118
eGFR<60 mL/min/1.73 m^2	1	486.744	<.0001	1	413.976	<.0001
Proteinuria[a]	1	2080.883	<.0001	1	1191.305	<.0001
Body mass index (kg/m^2)						
<18.5	1	0.116	0.7339	1	73.186	<.0001
≥25.0, <30.0	1	0.863	0.3529	1	48.230	<.0001
≥30.0	1	0.111	0.7387	1	99.475	<.0001
Smoking						
Ex-smoker	1	254.747	<.0001	1	42.068	<.0001
Smoker	1	2762.972	<.0001	1	1313.720	<.0001
Pack-year	1	1013.516	<.0001	1	18.479	<.0001
Alcohol drinking						
Low risk	1	1867.009	<.0001	1	0.681	0.4092
Moderate risk	1	487.998	<.0001	1	20.558	<.0001
High risk	1	243.018	<.0001	1	11.498	0.0007
Activity group						
Moderate activity	1	379.096	<.0001	1	254.878	<.0001
High activity	1	221.749	<.0001	1	139.094	<.0001
Current medication						
Blood glucose-lowering drugs	1	2092.561	<.0001	1	1998.526	<.0001
Antihypertensive drugs	1	917.405	<.0001	1	1018.164	<.0001
Lipid-modifying drugs	1	4.225	0.0398	1	225.756	<.0001
Family history of heart	1	492.181	<.0001	1	15.111	0.0001

disease						
Family history of stroke	1	149.586	<.0001	1	149.586	<.0001

BP, blood pressure; HDL, high-density lipoprotein cholesterol; LDL, low-density lipoprotein cholesterol; eGFR, estimated glomerular filtration rate

[a]≥1+ in urine dipstick for proteinuria.
